# Differences in Body Composition Analysis by DEXA, Skinfold and BIA Methods in Young Football Players

**DOI:** 10.3390/children9111643

**Published:** 2022-10-28

**Authors:** José Francisco Tornero-Aguilera, Bella Esperanza Villegas-Mora, Vicente Javier Clemente-Suárez

**Affiliations:** 1 Faculty of Sports Sciences, Universidad Europea de Madrid, Tajo Street, s/n, 28670 Madrid, Spain; 2 Studies Centre in Applied Combat (CESCA), 45007 Toledo, Spain

**Keywords:** body fatness, body composition, skinfold thickness, bioelectrical impedance analysis (BIA), dual energy, X-ray absorptiometry (DEXA)

## Abstract

The most widely used method in professional sports for fat percentage assessment is the skinfold method. However, there is the chance of bias and human error. For this reason, other more precise methods are used, such as single-frequency bioelectrical impedance analysis (BIA) or dual energy X-ray absorptiometry (DEXA). However, there are limited data that demonstrate the methodological shortcomings or congruences in fat and fat-free mass estimates including gender differences and differences in athlete populations. Thus, the aim of the present study was to compare total body fat (%BF) estimated by six skinfold thickness measurement (SKF) and single-frequency bioelectrical impedance analysis (BIA) methods, using three different sets of equations, to that assessed by the dual energy X-ray absorptiometry (DEXA) method using a DEXA Hologic Serie Discovery QDR. For this aim, 76 males and 70 females belonging to the professional Spanish football federation were evaluated. We found significant differences between the three measures. BIA significantly underestimates the fat percentage, followed by skinfolds. With DEXA being the more objective or accurate method, an equation is established by means of linear regression analysis that allows the percentage of adipose tissue to be obtained either through anthropometry or electrical bioimpedance and adjusted to that which would be obtained by the DEXA system.

## 1. Introduction

From health to performance, body fat percentage (%BF) is a major issue. The population trend has shown an increased tendency toward overweight and obesity, estimating that twenty-three percent of the world’s adult population is pre-obese and nine percent is obese, with over 50% of the European adult population being pre-obese or obese [1]. Knowing that adipose tissue and the inflammatory cytokines that it produces are directly related to the development of most Western diseases [2], its quantification and measurement in intervention processes are essential. Authors suggest that adipocyte overproduction of TNF-alpha and several interleukins (IL), notably IL-1beta and IL-6, are behind the slowdown in physical recovery [3]. Likewise, in sports performance, athletes with greater muscle mass and less BF can cover longer distances per match [4], obtain higher values in jumping [5], acceleration or resistance tests, and can facilitate a high-intensity intermittent performance for prolonged periods [6]. In fact, recent reviews affirm that there is a directly proportional relationship between a higher adipose percentage and lower sports performance [7]. Furthermore, %BF is considered as a great longitudinal predictor for leg power in young soccer players, and the routine evaluation of %BF throughout the season provides useful information for coaches and nutritionists to monitor the efficacy of training and nutrition interventions [8].

Among the different methods for the quantification and estimation of BF after underwater weighing, the most used is based on the sum of four skinfold thicknesses according to Durnin and Womersley (SKF) [9]. We also found the bioelectrical impedance analysis (BIA) and finally dual energy X-ray absorptiometry (DEXA) methods. These methods are different not only in the principles of measurements, but also in the assumptions required for the calculations and the prediction equations. Thus, there are methodological shortcomings or incongruence in fat and fat-free mass estimation. In this line, the DEXA system is considered the most objective and accurate dispositive among them and one of the reference methods for measuring BF%. This is mainly due to the long-term precision of the values due to the coefficient of variation (CV) of repeated measurements of 2% [10]. However, its use is limited to clinical settings, and it is difficult to use in the field. Likewise, the scientific literature has mainly focused on healthy or pathological populations, but not athlete populations. Regarding BIA systems, these are methods that noninvasively allow BF% measurements with advantages over DEXA of portability, simplicity, speed, safety, and low cost. However, measurements are conditioned to sex, age, ethnicity, level of fat percentage, hydration level, and the day and hour time of measurement [11]. This requires systematic conditions for a correct evaluation. Likewise, it is suggested that this system may include misinterpretations of the results in bulky people or those with high adipose tissue, explained by their altered body geometry and body water distribution [12].

On the other hand, SKF is postulated as a validated tool with the DEXA system for the calculation of BF% and as an excellent field tool. However, the risk of bias is present since the measurements depend greatly on the skill and training of the subject when taking the skinfolds. Likewise, there are several equations for calculating the %BF, among which are two equations that have been validated, that proposed by Slaughter et al. (1988) [13] mainly used in a healthy nonathletic population, and the recent soccer-specific equation proposed by Munguia-Izquierdo et al. (2018) [14], which is the one used in the present study.

Currently, there are a few studies that have reported mixed findings in validating the BIA, DEXA analyzers and skinfold thickness equations as methods for measuring BF%. Of the most cited studies, either the sample is small, or they do not consider gender differences [10,15,16,17]. Furthermore, the use of the DEXA system is expensive and not accessible to everyone. Thus, the present research has the aims of (i) comparing body fat percentage with DEXA, BIA and SKF parameters in trained male and female football players; (ii) establishing two equations by linear regression analysis to obtain the percentage of adipose tissue by anthropometry and electrical bioimpedance adjusted by the DEXA measurements. The initial hypothesis was that there would be differences between the three body composition analysis methods.

## 2. Materials and Methods

We analyzed 146 football players (76 males and 70 females) belonging to the Spanish football federation. The physical characteristics of the football players are shown in Table 1. The data were extracted during the week prior to the start of the preseason, together with the completion of the rest of the routine medical tests. The research procedure was conducted following the Declaration of Helsinki (revised in Brazil, 2013) and approved by the Ethics Committee of the European University (CIPI/21/082 date of approval 29 April 2021). Before starting the study, all participants were informed about the process to be carried out and gave their voluntary written informed consent.

Present study aim is to analyze differences in body composition parameters with direct (DEXA) and indirect (BIA and SKF) measurements in trained football players, using standardized procedures as in previous researches [8]. The %BF estimations from BIA measurements and anthropometric equations during the preseason were compared with DEXA results to determine the sensitivity to change of these practical methods for use in trained football players.

Participants were instructed to follow an identical assessment session (between 8:30 a.m. and 10.50 a.m.) in the same well-ventilated room with controlled temperature and humidity (22.4 ± 0.8 °C, and 30.2 ± 2.1% of humidity), following standard food and fluid protocols. Thus they were: rested, overnight fasted, hydrated and with an empty bladder and bowel before testing. During the evaluation, participants only wore shorts and were asked to remove any metal and jewelry. The inclusion criteria were belonging to the professional Spanish football federation, while exclusion criteria were use of ergogenic aids, strenuous exercise performance, or alcohol, stimulants or depressants consumption 2 days prior to the evaluation.

### 2.1. Anthropometry Measurements

Body height and weight were measured with a SECA model 714 Hamburg, Germany.

Regarding body mass index (BMI), the formula body mass (kg)/height (m^2^) was used. Then, the calf, thigh, waist, hip, arm relaxed and arm flexed circumferences were measured. After the circumferences the eight skinfold of medial calf, anterior thigh, iliac crest, abdominal, subscapular, supraspinal, biceps and triceps were measured. Caliper, tape and skinfold caliper (Holtain, Crymych, United Kingdom) were used, following previous research [8]. Three measures of each anthropometric variable were taken. Fat percentage was obtained with the soccer-specific equation of Munguia-Izquierdo et al., 2018 [14]. The same instructed professional nutritionist with experience in sampling athletes conducted this procedure.

### 2.2. Bioelectrical Impedance Analysis

Prior to analysis feet soles and hand palms were wiped with an electrolyte tissue provided by InBody. Then an InBody 770 Biospace, Seoul, South Korea, was used. This is a multifrequency impedance plethysmograph body composition analyzer, which takes readings from the body using an 8-point tactile electrode method, measuring resistance at 5 specific frequencies (1, 50, 250, 500 kHz and 1 MHz) and reactance at 3 specific frequencies (5, 50 and 250 kHz) on each of 5 segments (right arm, left arm, trunk, right leg and left leg) [8]. To carry out the tests, the subjects stood upright on foot electrodes on the instrument platform, with legs and thighs apart and arms not touching the torso. They were barefooted and without excess clothing. Four foot electrodes were used, two of which were oval-shaped and two heel-shaped, and prior to testing both the skin and the electrodes were cleaned and dried [18].

### 2.3. Dual Energy X-ray Absorptiometry

DEXA Hologic Serie Discovery QDR, Software Physician’s Viewer, APEX System Software Version 3.1.2. Bedford, MA, USA was used. Prior to the start of evaluations, a rutinary preliminary analysis was made with the manufacturer’s phantom. The same certified technician performed all the scans, executing the evaluations in routine clinical manner following research facility standard operating procedures. For the analysis, all scans used the auto-analysis feature. Only manual correction analysis was performed when necessary to avoid possible errors. Whole body scan and regions were analyzed in a routine and clinical way following manual and recommendations of authors [19].

### 2.4. Statistical Analysis

The SPSS statistical package (version 21.0; SPSS, Inc. Chicago, IL, USA) was used to analyse the data. Normality and homogeneity were tested using the Shapiro–Wilk test and Levene’s test, respectively. Group comparisons were performed with parametric or nonparametric tests. We used the *t* test for independent samples or the Mann–Whitney U for group comparisons (i.e., females vs. males). The results of %BF with DEXA were compared with other methods using the one sample *t* test. Furthermore, Bland–Altman diagrams were presented to test agreement between methods. Regression equations were developed for estimating %BF regarding the DEXA method using data of anthropometric measurements and bioimpedance. We analysed coefficients of determination (i.e., R2 and AdjR2) and standard errors of estimation (SEE). The level of significance for all the comparisons was set at *p* < 0.05.

## 3. Results

The results are reported with their mean and standard deviation. Table 1 shows demographic and anthropometric, bioelectrical impedance and dual energy X-ray absorptiometry data distributed by gender.

**Figure 1:**
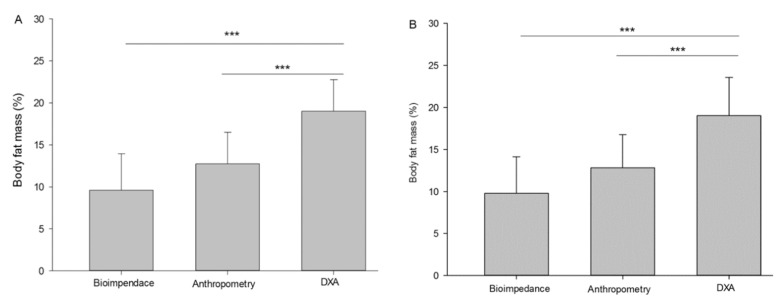
Body fat mass (%) in male (**A**) and female (**B**) soccer players. *** = *p* < 0.001.

Figure 1 introduces a comparison between the three measurement methods, anthropometric, bioelectrical impedance, and dual energy X-ray absorptiometry, in relation to the percentage of fat. Both methods are segmented by gender, male (Panel A) and female (Panel B). The level of statistical significance was established at *p* < 0.001.

**Figure 2:**
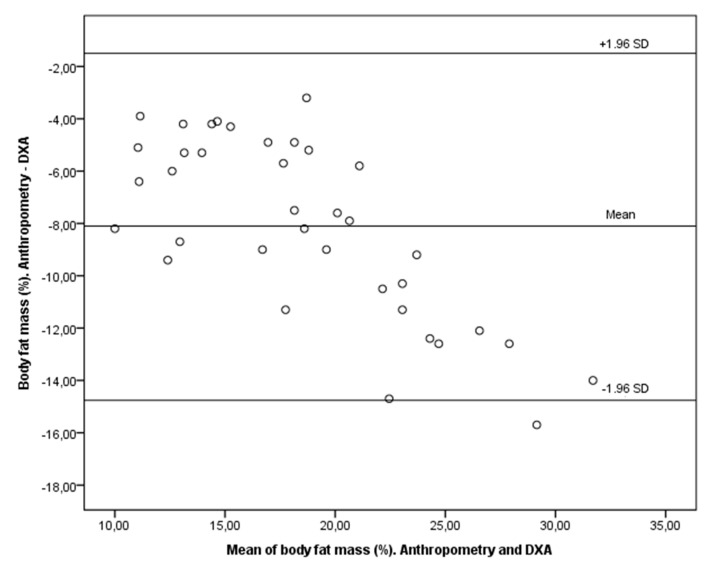
Bland–Altman plot analysis for body fat mass (%) with anthropometry and DEXA. *p* = 0.000.

**Figure 3:**
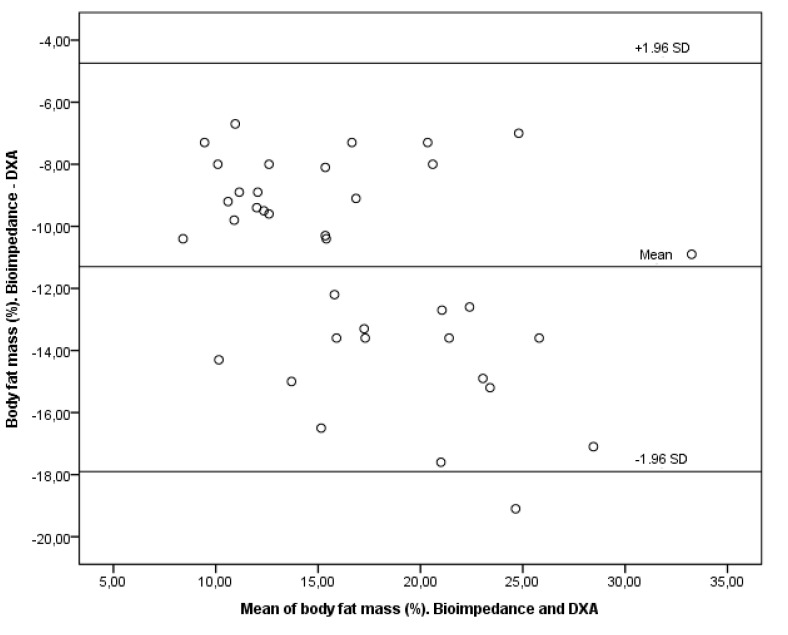
Bland–Altman plot for body fat mass (%) with bioimpedance and DEXA for all participants. *p* < 0.001.

The following figures are a Bland–Altman plot analysis showing the limits of agreement between paired values for body fat mass (%) with anthropometry and DEXA for all participants (Figure 2) and a Bland–Altman plot showing the limits of agreement between paired values for body fat mass (%) with bioimpedance and DEXA for all participants (Figure 3), both with a significance of *p* < 0.001.

Furthermore, a linear regression analysis was carried out and the following equations were obtained. The equations allow the percentage of adipose tissue to be obtained, either through anthropometry or electrical bioimpedance and adjusted to that which would be obtained through the DEXA system.

Fat mass_DEXA (%) = 3.62 + (1.326 × Fat mass_Anthropometry%); R = 0.909 Rsqr = 0.826 Adj Rsqr = 0.821 and Standard Error of Estimate = 2.707.

Fat mass_DEXA (%) = 10.947 + (1.030 × Fat mass_Bioimpedance%); R = 0.877 Rsqr = 0.770 Adj Rsqr = 0.763 and Standard Error of Estimate = 3.113.

## 4. Discussion

The present research has the aims of (i) comparing body fat percentage with the DEXA, BIA and SKF parameters in trained male and female football players; (ii) establishing two equations by linear regression analysis to obtain the percentage of adipose tissue by anthropometry and electrical bioimpedance adjusted by the DEXA measurements. Body fat percentage measurement results were as follows in males: DEXA (19.0 ± 3.7), BIA (9.3 ± 4.3) and SKF (12.7 ± 3.7), and in females: DEXA (29.2 ± 4.8), BIA (14.9 ± 5.6) and SKF (17.8 ± 3.7), showing clear differences between measuring methods. As such, our hypothesis cannot be confirmed. This correlates with a previous study, which also showed clear differences in SKF and DEXA measurements in 238 Caucasian adolescents [20], while another study showed agreement between both procedures in 42 African American and Caucasian women [21]. Furthermore, Lozano Berges et al. (2017) [22] compared DEXA, BIA, SKF and air displacement plethysmography in young male and female soccer players (13.4 ± 0.6 years) concluding that these methods for body composition measuring are not comparable in this sample. On this line, the development of an equation that allows the extrapolation of adipose tissue percentage as measured by bioimpedance and anthropometry to that obtained through DEXA would be a very useful achievement for professionals all around the world.

In addition, body composition analysis is essential to control high-level sport athletes since it is a major health and sport-related physical fitness component, directly related to performance. In sport sciences, the fourth level (tissues–systems) is evaluated. According to it, the human body is divided into fat mass and fat-free mass. In this line, recent studies suggest that players’ overall mean percentage values of body fat vary between 9.9 and 11.9% for elite males and between 12.4 and 16.5% for amateur senior soccer players [23]. In female players, the average body fat for an amateur is 25.9 ± 2.0% [24] and it is 19.7 ± 0.7 for elite players [25]. According to our data, very similar results were seen since females presented with around 29.2% body fat and males with 19% when measured with DEXA. Likewise, there is a trend in which the fat percentage values are higher in the measurement with DEXA than those with BIA or skinfolds. In this line, similar results and trends have been observed in other international-level elite soccer players, such as in the Italian Serie A league [26], Australian A-league [27], English Premier league [28] or even the Spanish league [29]. The differences found may be mainly due to the previous physical condition of the players, the sport’s history, age and ethnicity, the league to which they belong and their experience and previous training [23,30]. Additionally, differences between genders are produced by the different physiologies of each gender, with females typically presenting ~10% higher body fat percentage compared to men because of the additional fat accumulation preparing females for a possible pregnancy [31]. Furthermore, differences could be attributed to the different DEXA model used and its software [29].

Furthermore, regarding the sum of skinfolds, it presents a high correlation with DEXA [29] in both males and females, which is consistent with the findings of previous studies in male soccer players [26,32]. In women, to our knowledge, this is one of the first studies to show this positive correlation. Thus, the skinfold measurement is a great option to estimate the %BF in either male or female players. Indeed, UEFA’s nutritional expert group suggests it as an effective, reliable and replicable tool, as long as the evaluator is well trained [33]. Additionally, BIA is considered and recognized as a validated method for body composition analysis [34]. However, it is limited when estimating %BF. The authors suggest that BIA analysis is reasonable in controlled conditions and in healthy and euvolemic adults, but not as accurate in people of great volume [35] or athletes [36]. Indeed, our results suggest that BIA has the lowest correlation with the DEXA method, significantly underestimating the %BF. Compared to the skinfold methods, although the BIA method is faster, portable and validated, it does not seem to be the best option for this population group, which is consistent with the findings of other studies in soccer players [26,29].

DEXA is considered to be the more objective and accurate dispositive method for assessing fat mass in athletes. However, the equipment is costly and not accessible for many institutions, medical sport centers or clubs. Thus, field methods such as anthropometry and BIA are used to evaluate body composition in athletes. In this line, and to obtain similar results as with the DEXA scan, a linear regression analysis was carried out and the equations previously mentioned were obtained. The equations allow the percentage of adipose tissue to be obtained either through anthropometry or electrical bioimpedance and adjusted to that which would be obtained through the DEXA system. To obtain the DEXA adipose percentage values measured with skinfolds, the equation used would be the following: 3.362 + (1.326 × fat mass anthropometry%); for BIA 10.947 + (1.030 × fat mass bioimpedance%). This equation will allow evaluators who do not have a DEXA system to adjust the fat percentages found with other methods. In this way, the monitoring and control, not only of the weight but also of the percentage of adipose tissue, so essential in sports, becomes more accurate and objective.

## 5. Practical Applications

The formulas obtained in the present research would allow for more accurate control and evaluation of the body composition of a football player, facilitating the analysis of body composition with cheaper systems, and easy transport, and thus making the field evaluations more efficient and effective, being able to provide stricter control of the athletes.

## 6. Future Lines of Research and Limitation of the Study

Future lines of research could try to analyze different collective sports such as basketball, volleyball, hockey or handball, and to analyze differences by age groups. Another research line would be to compare with the gold standard bod pod or underwater weight.

As a limitation of the study, we can highlight the low number of participants, but at the time of the analysis, access to a large sample was limited. Another limitation could be the possible human error when taking the data with the different measurement systems.

## 7. Conclusions

We found significant differences between the three measures. BIA significantly underestimates the fat percentage, followed by skinfolds. With DEXA being the more objective and accurate method, an equation is established by means of linear regression analysis that allows the percentage of adipose tissue to be obtained either through anthropometry or electrical bioimpedance and adjusted to that which would be obtained by the DEXA system.

## Figures and Tables

**Table 1:** Characteristics of participants.

Variables	Females (n = 70)	Males (n = 76)	Statistical Significance
Age (yrs)	22.3 ± 3.2	21.8 ± 5.0	*p* > 0.05
Stature (cm)	162.1 ± 6.5	175.0 ± 7.1	*p* < 0.001
Weight (kg)	59.6 ± 8.3	71.4 ± 7.1	*p* < 0.001
BMI (kg/m^2^)	22.7 ± 2.6	23.3 ± 2.1	*p* > 0.05
Fat mass (%). Bioimpedance	14.9 ± 5.6	9.3 ± 4.3	*p* < 0.01
Fat mass (%). Anthropometry	17.8 ± 3.7	12.7 ± 3.7	*p* < 0.001
Fat mass (%). DEXA	29.2 ± 4.8	19.0 ± 3.7	*p* < 0.001
∑6 skinfold measurements	91.8 ± 23.7	61.7 ± 20.8	*p* < 0.001
∑8 skinfold measurements	117.7 ± 29.1	81.0 ± 27.5	*p* < 0.001

## Data Availability

All the data are in the manuscript.
